# Computational Estimation of the Acidities of Pyrimidines and Related Compounds †

**DOI:** 10.3390/molecules27020385

**Published:** 2022-01-07

**Authors:** Rachael A. Holt, Paul G. Seybold

**Affiliations:** Department of Chemistry, Wright State University, Dayton, OH 45435, USA; rachaelannholt@gmail.com

**Keywords:** pyrimidines, acidities, pK_a_, density-functional theory, QSAR

## Abstract

Pyrimidines are key components in the genetic code of living organisms and the pyrimidine scaffold is also found in many bioactive and medicinal compounds. The acidities of these compounds, as represented by their pK_a_s, are of special interest since they determine the species that will prevail under different pH conditions. Here, a quantum chemical quantitative structure–activity relationship (QSAR) approach was employed to estimate these acidities. Density-functional theory calculations at the B3LYP/6-31+G(d,p) level and the SM8 aqueous solvent model were employed, and the energy difference ∆E_H2O_ between the parent compound and its dissociation product was used as a variation parameter. Excellent estimates for both the cation → neutral (pK_a1_, R^2^ = 0.965) and neutral → anion (pK_a2_, R^2^ = 0.962) dissociations were obtained. A commercial package from Advanced Chemical Design also yielded excellent results for these acidities.

## 1. Introduction

Pyrimidines play a central role in the terrestrial genetic code and the pyrimidine framework is also found in many bioactive compounds and medicines. As measurements of molecular properties are frequently difficult or expensive, it is of interest to develop theoretical means for estimating these properties. Among the most important and interesting properties of the pyrimidines are their acidities, as represented by their pK_a_ values, which determine the forms of the compounds that will prevail in solution under different pH conditions. As a result, there has been a long-standing interest in estimating the pK_a_s of chemical compounds using theoretical approaches [1,2]. This interest continues, as demonstrated by the broad range of methods employed in recent pK_a_ studies [3,4,5,6,7,8,9,10]. In an earlier study by our group [11], we presented computational estimates of the pK_a_s of the biologically related purines and indoles. In the present work we develop estimates for the acidities of pyrimidines and related compounds.

Three main approaches have been used in studies estimating compound acidities [1,12]. In the first approach, *first-principles* or *absolute* pK_a_s are determined in a straight-forward manner using direct calculations, often relying on a thermodynamic cycle to separate different hypothetical stages [13,14]. The advantage of this approach is that it follows standard procedures and does not depend on prior knowledge of experimental results. Its disadvantage is that it normally requires a high level of computational effort to achieve reasonable accuracy. A second approach employs the development of an appropriate *Quantitative Structure–Activity Relationship* (QSAR) for the acidities of a set of compounds [2,15]. This approach requires first the collection of measured experimental pK_a_ values for the set and then the identification of some suitable molecular parameter that is closely related to the acidity. An advantage of this approach is that it can generally achieve high accuracy while employing more modest computational efforts, and it accordingly allows the estimation of the pK_a_s of related, unreported compounds using the same modest computational effort. Furthermore, digressions from the derived regression algorithms (i.e., outliers) can alert practitioners to compounds that may be exhibiting behaviors different from the others in the sample [16,17,18]. In a third approach, a number of commercial programs use algorithms derived from large acidity databases and employ empirical parameters such as Hammett constants to estimate pK_a_s [19,20]. The latter two approaches were employed in the present study.

As noted, the QSAR approach relies on the discovery of some parameter or property of the compounds examined that correlates with the activity of interest, in the present case the pK_a_. In many cases, partial atomic charges have been employed as variation parameters for acidities and other properties. However, because the notion of an “atomic charge” in a molecule is not a proper quantum chemical observable, a variety of different approximate schemes have been developed to represent these atomic charges. Of these proposed schemes, our own group [21,22,23,24] and others [5,25,26,27] have found the natural population analysis (NPA) orbitals and charges developed by Weinhold et al. [28,29,30] to be especially useful in this role. More recently, we have also used another parameter, the energy difference ∆E_H2O_ between the parent compound and its main dissociation product for this purpose. It is this parameter that was employed in the present study.

A particular difficulty arises in a study of the pyrimidines because these compounds do not typically appear as a single species in gas phase or solution, but rather are present as a collection of related tautomers, a situation that also prevailed in our earlier study of purines [11]. Accordingly, some accommodation must be made for this condition, as will be described in the following section.

## 2. Methods

Our aim in a recent series of reports has been to design and evaluate a QSAR protocol suitable for producing accurate pK_a_ estimates for selected classes of compounds while employing relatively modest and commonly available computational tools.

The initial step in a pK_a_ QSAR study involves the collection of reported experimental pK_a_ values from the literature for the class of compounds of interest. In the present case, values for both the cation-to-neutral dissociation, AH_2_^+^ → AH + H^+^, which we will designate pK_a1_, and the neutral-to-anion dissociation, AH → A^−^ + H^+^, which we will call pK_a2_, were available for a number of pyrimidines and related compounds. These values are tabulated in Table 1. Given in Table 1 also are computed pK_a_ values obtained from the Advanced Chemical Development (ACD) [31] commercial software package.

Calculations were carried out using the Spartan’10 software package (Wavefunction, Inc., Irvine, CA). In earlier pK_a_ studies, we found that density-functional computations at the B3LYP/6-31+G(d,p) level provided accurate accounts of molecular properties while still requiring only modest computational demands. After testing this assumption (*vide infra*) against available gas-phase experimental results for pyrimidines in the NIST database [40], this level of theory was adopted in the present work. For the studies in aqueous solution, the SM8 aqueous solvent model of Marenich et al. [41,42] was used. This same solvent model was also employed in our earlier study of purines and indoles [11] and other studies [16,17,43], where it was shown to perform well in helping to reproduce the experimental data.

As noted above, many of the compounds examined here exist in several tautomeric forms in solution. (For example, uracil has three cationic tautomers, six neutral tautomers, and two anionic tautomers.) Accordingly, the relative stabilities of the tautomeric forms—cationic, neutral, and anionic—of each compound were evaluated within the SM8 aqueous solvent model, and the most stable tautomer for each condition was taken as representative of that compound for computational purposes [44]. Fortunately, there frequently exists a substantial energy gap (>25 kJ/mol) between the most stable tautomer and the remaining tautomers of that species; however, ultimately the validity of this approximation must await justification from the results of the subsequent analysis. In earlier studies, we found that for neutral → anion dissociations (pK_a2_) the value ∆E_H2O_ = E_H2O_(A^−^) − E_H2O_(AH) for the energy difference between the parent compound AH and its dissociation product A^-^ in aqueous solution provides an excellent regression parameter for QSAR pK_a_ studies, and after testing several other parameters this descriptor was used in the present studies. For the cation → neutral dissociation (pK_a1_), the analogous expression ∆E_H2O_ = E_H2O_(AH) − E_H2O_(AH_2_^+^) was used.

We also provide a note of caution regarding directly comparing numerical results found here using *Spartan’10* with those obtained using the popular *Gaussian* computational package (Gaussian, Inc., Wallingford, CT 06492, USA), since these programs use different basis sets for some atoms [18,45].

## 3. Results and Discussion

We first wished to assure that the B3LYP/6-31+G(d,p) level of computation was sufficient to provide accurate results for the dissociations in question. For this, we turned to gas-phase reaction results reported in the NIST chemical database [24]. This database contains gas-phase thermodynamic data for the Gibbs energy change ∆_r_G° for the anion + H^+^ → neutral reaction, for six of the compounds studied here. These experimental data are compared with our computed ∆_r_G° values in Table 2 (note that G°(H^+^) is −26.3 kJ/mol at 298.15 K [1]). As can be seen, there is very close agreement between the experimental and calculated Δ_r_G° values. With the exception of succinimide, all of the calculated values fall within the estimated experimental errors given in the NIST database. This, and the coefficient of determination of R^2^ = 0.998 between the computed and experimental values, suggest that the level of computation described should provide a good account of the reactions to be considered.

As noted above, in previous studies we have found that the energy difference ΔE_H2O_ between the parent compound and its dissociation product provides an excellent regression variable for pK_a_ QSAR estimations. We first examined employment of the gas-phase ΔE values for this purpose. As expected, the use of ΔE_gas_ values yielded good, but not exceptional, correlations for both the pK_a1_ (cation → neutral, R^2^ = 0.707) and pK_a2_ (neutral → anion, R^2^ = 0.874) dissociations.

We next optimized the compounds within the SM8 aqueous solvent model. Using the solvent-optimized structures and the calculated ∆E_H2O_ values for the appropriate reactions, we obtained the following QSAR models:

pK_a1_ (calc.) = −0.131 (± 0.008) · ∆E_H2O_ − 151.54 (± 10) (1)

n = 17, R^2^ = 0.965, s = 1.25, F = 145

pK_a2_ (calc.) = −0.141 (± 0.008) · ∆E_H2O_ − 159.42 (± 10) (2)

n = 12, R^2^ = 0.962, s = 0.612, F = 304

In these equations, n = the number of compounds in the sample, R^2^ is the coefficient of determination (the fraction of the variance in the data accounted for by the model), s is the standard error of the estimate, and F is the Fisher statistic. It is evident that optimization of the structures within the solvent model significantly increases the accuracy of the model, as was also shown in earlier work [1,7,8,9].

**Figure 1:**
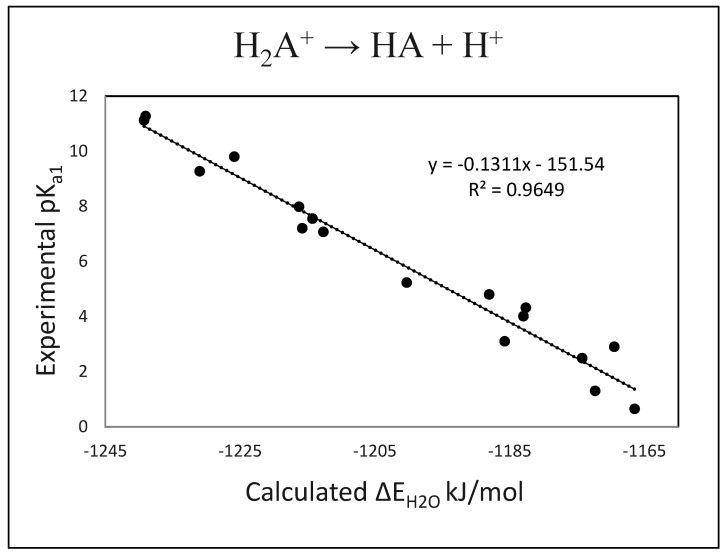
Plot of experimental pK_a1_s vs. calculated ΔE_H2O_s.

**Figure 2:**
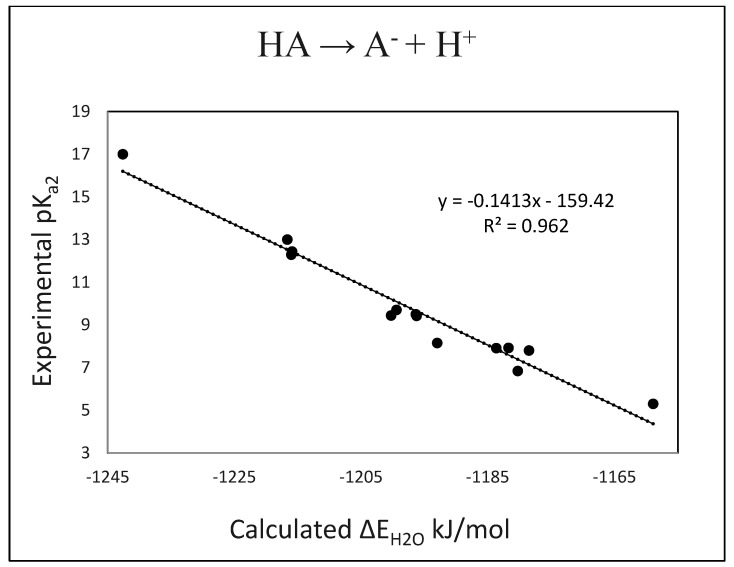
Plot of experimental pK_a2_s vs. calculated ΔE_H2O_s.

The results for pK_a1_ and pK_a2_ are plotted in Figure 1 and Figure 2, and the calculated values are compared with the experimental values in Table 3 and Table 4. We note that several of the pK_a_s for the cation → neutral dissociation (pK_a1_) fall into the difficult-to-measure negative value range and carry large uncertainties. Accordingly, these values are also not well characterized for use in this range in forming the regression Equation (1), and we prefer to recognize this uncertainty by simply indicating modestly negative (<0) or significantly negative (<<0) for the pK_a_s of these compounds.

We also tested the ability of a commercial software program, Advanced Chemical Development, Inc.’s ACD/Labs PhysChem Percepta Suite, to estimate these pK_a_s. The results for pK_a1_ showed an excellent correlation:

pk_a1_ (exp.) = 1.07 (±0.03) ∙ pk_a1_ (ACD) + 0.34 (±0.2) (3)

where n = 19, R^2^ = 0.991, s = 0.334, and F = 1583.

The results for pK_a2_ were also very good:

pK_a2_ (exp.) = 0.95 (±0.05) ∙ pK_a1_ (ACD) + 0.92 (±0.52) (4)

where n = 13, R^2^ = 0.966, s = 0.612, and F = 316.

These results encourage use of this software for studies of the pK_a_s of these compounds.

## 4. Conclusions

A primary endeavor of scientific studies is to develop models of physical, chemical, and biological systems for the purpose of understanding these systems better. All models are by their very nature approximate. However, as Gauch has noted [46], in some cases—counterintuitively—a model can be more accurate than the data from which it is constructed “because it amplifies hidden patterns and discards unwanted noise” inherent in the system being examined. The QSAR equations used here take advantage of this property by “averaging through” the noise, or random errors, in the experimental pK_a_ data. It is evident that both the QSAR Equations (1) and (2) above provide relatively simple means, via mathematical *models*, for estimating the pK_a_s of the pyrimidines and related compounds. For example, in order to estimate the pK_a_s of unmeasured compounds in this class or to check the reported pK_a_s of measured compounds, using the QSAR equations one merely needs to determine ∆E_H2O_ for the compound and then evaluate the pK_a_ from the appropriate regression equation. Therefore, the equations provided should allow reasonable estimations for the pK_a_s of other pyrimidines and compounds similar to the pyrimidines. Use of the commercial ACD/Labs program can provide a further independent and very useful check on the pK_a_ estimates.

## Figures and Tables

**Table 1:** Reported experimental pK_a1_ and pK_a2_ values and ACD computed values for the pyrimidines and related heterocycles studied.

No.	Compound	Formula	pK_a1_	ACD pK_a1_	pK_a2_	ACD pK_a2_
1	azauracil	C_3_H_3_N_3_O_2_	-	−4.4 ± 0.2	-	7.8 ± 0.2
2	aziridine	C_2_H_5_N	7.98 [32], 8.05 [33]	8.1 ± 0.2	-	-
3	creatinine	C_4_H_7_N_3_O	4.8 [34]	4.8 ± 0.1	-	-
4	cytosine	C_4_H_5_N_3_O	4.32 [30], 4.58 [35], 4.6 [31]	4.4 ± 0.1	13 [33], 12.15 [36]	12.3 ± 0.1
5	flucytosine	C_4_H_3_FN_2_O_2_	3.26 [34]	2.6 ± 0.1	-	10.5 ± 0.1
6	imidazole	C_3_H_4_N_2_	7.15 [33], 6.99 [34,35]	7.2 ± 0.6	14.44 [33]	13.9 ± 0.1
7	1-methylimidazole	C_4_H_6_N_2_	6.95 [34]	7.0 ± 0.1	N/A	-
8	4-methylimidazole	C_4_H_6_N_2_	7.55 [35]	7.7 ± 0.6	-	14.3 ± 0.1
9	isocytosine	C_4_H_5_N_3_O	4.01 [34]	3.4 ± 0.5	9.42 [36]	9.6 ± 0.4
10	isoxazole	C_3_H_6_NO	−2.0 [34]	−2.0 ± 0.5	N/A	
11	maleimide	C_4_H_3_NO_2_	-	−5.7 ± 0.2	9.46 [37]	8.5 ± 0.2
12	morpholine	C_4_H_9_NO	8.492 [35]	9.0 ± 0.2	N/A	-
13	piperidine	C_5_H_11_N	11.12 [35]	10.4 ± 0.1	-	-
14	piperazine	C_4_H_10_N_2_	9.78 [35], 9.73 [34]	9.6 ± 0.1	-	-
15	1-methylpiperazine	C_5_H_12_N_2_	10.19 [35]	9.3 ± 0.1	-	-
16	oxazole	C_3_H_6_NO	0.8 [34]	1.0 ± 0.1	N/A	-
17	pyrazine	C_4_H_4_N_2_	0.65 [34]	1.2 ± 0.1	N/A	-
18	pyrazole	C_3_H_4_N_2_	2.61 [35]	2.8 ± 0.1	14.21 [33]	14.0 ± 0.5
19	pyridazine	C_4_H_43_N_2_	2.3 [32]	3.1 ± 0.1	N/A	-
20	pyridine	C_5_H_5_N	5.23 [34]	5.2 ± 0.1	N/A	-
21	pyrimidine	C_4_H_4_N_2_	1.3 [32]	1.8 ± 0.1	N/A	-
22	pyrrole	C_4_H_5_N	−3.8 [34]	−0.3 ± 0.5	17.0 [33]	17.0 ± 0.5
23	pyrrolidine	C_4_H_9_N	12.10 [33], 11.31 [34,35]	10.5 ± 0.1	-	-
24	succinimide	C_4_H_5_NO_2_	-	−4.4 ± 0.2	9.62 [34,35], 9.68 [36]	9.6 ± 0.1
25	thymine	C_5_H_6_N_2_O_2_	-	−4.1 ± 0.4	9.9 [33], 9.79 [35], 9.44 [34]	9.2 ± 0.1
26	uracil	C_4_H_4_N_2_O_2_	-	−4.2 ± 0.1	9.43 [38], 9.45 [34,36]	8.9 ± 0.1
27	5-bromouracil	C_4_H_3_BrN_2_O_2_	-	-	7.91 [38]	6.8 ± 0.1
28	5-chlorouracil	C_4_H_3_CIN_2_O_2_	-	-	7.92 [38]	6.8 ± 0.1
29	fluorouracil	C_4_H_4_FN_3_O	-	-	8.04 [30], 8.00 [39], 7.93 [38]	6.7 ± 0.1
30	5-formyluracil	C_5_H_4_N_2_O_3_	-	-	6.84 [38]	7.3 ± 0.1
31	5-nitrouracil	C_4_H_3_N_3_O_4_	-	-	5.3 [38]	5.2 ± 0.1

**Table 2:** Experimental and calculated gas-phase Δ_r_G° values (kJ/mol) for the reaction A^−^ + H^+^ → AH.

Compound	Exp. Δ_r_G° ^a^	Calc. Δ_r_G° ^b^	Calc. ΔE ^b^
pyridine	1601	1605	1648
pyrazine	1605	1605	1643
pyrimidine	1577	1579	1614
pyridazine	1565	1562	1601
imidazole	1433	1432	1466
succinimide	1414	1401	1436

^a^ From the NIST database, Ref. [40]; ^b^ B3LYP/6-31+G(d,p).

**Table 3:** Literature pK_a1_s and estimated pK_a1_s.

Compound	ΔE kJ/mol	Exp. pK_a1_	Calc. pK_a1_ ^a^	Residual
azauracil	−1084	-	<<0	-
aziridine	−1216	8.01	7.76	0.25
creatinine	−1188	4.8	4.09	0.71
cytosine	−1183	4.5	3.43	1.07
flucytosine	−1169	3.26	1.60	1.66
imidazole	−1213	7.07	7.36	−0.29
1-methylimidazole	−1216	7.95	7.76	0.19
4-methylimidazole	−1215	7.55	7.63	−0.08
isocytosine	−1183	4.01	3.43	0.58
isoxazole	−1115	−2	<0	-
maleimide	−1035	-	<<0	-
oxazole	−872	0.8	<<0	-
piperidine	−1239	11.12	10.77	0.35
piperazine	−1226	9.76	9.07	0.69
1-methylpiperazine	−1231	10.19	9.72	0.47
pyrazine	−1166	0.65	1.21	−0.56
pyrazole	−1174	2.61	2.25	0.36
pyridazine	−1186	2.3	3.83	−1.53
pyridine	−1200	5.23	5.66	−0.43
pyrimidine	−1172	1.3	1.99	−0.69
pyrrolidine	−1239	11.71	10.77	0.94
succinimide	−1007	-	<<0	-

^a^ pK_a1_s were estimated using Equation (1).

**Table 4:** Literature pK_a2_s and estimated pK_a2_s.

Compound	ΔE kJ/mol	Exp. pK_a2_	Calc. pK_a2_ ^a^	Residuals
azauracil	−1179	-	6.82	-
aziridine	−1325	-	27.41	-
creatinine	−1217	-	12.18	-
cytosine	−1216	12.57	12.04	0.53
isocytosine	−1196	9.42	9.22	0.20
flucytosine	−1254	-	17.39	-
imidazole	−1216	14.4	12.04	2.36
4-methylimidazole	−1224	-	13.16	-
maleimide	−1196	9.5	9.22	0.28
piperazine	−1313	-	25.71	-
1-methylpiperazine	−1395	-	37.28	-
piperidine	−1310	-	25.29	-
pyrrole	−1243	17	15.84	1.16
pyrrolidine	−1390	-	36.57	-
thymine	−1199	9.71	9.64	0.07
uracil	−1200	9.44	9.78	−0.34
5-bromouracil	−1184	7.91	7.52	0.39
5-chlorouracil	−1182	7.92	7.24	0.68
fluorouracil	−1193	7.99	8.79	−0.80
5-formyluracil	−1180	6.84	6.96	−0.12
5-nitrouracil	−1159	5.3	4.00	1.30

^a^ pK_a2_s were estimated using Equation (2).

## Data Availability

See Ref. [44] for available data.

## Sample Availability

Samples of the compounds are not available from the authors.

## References

[1] Shields G.C., Seybold P.G. (2014). Computational Approaches for the Prediction of pK_a_ Values.

[2] Ho J. (2014). Predicting pK_a_ in Implicit Solvents: Current Status and Future Directions. Aust. J. Chem..

[3] Pracht P., Grimme S. (2021). Efficient Quantum-Chemical Calculations of Acid Dissociation Constants from Free-Energy Relationships. J. Phys. Chem. A.

[4] Morency M., Néron S., Iftimie R., Wuest J.D. (2021). Predicting p*K*_a_ Values of Quinols and Related Aromatic Compounds with Multiple OH Groups. J. Org. Chem..

[5] Haslak Z.P., Zareb S., Dogan I., Aviyente V., Monard G. (2021). Using Atomic Charges to Describe the pKa of Carboxylic Acids. J. Chem. Inf. Model..

[6] Thapa B., Raghavachari K. (2019). Accurate pKa Evaluations for Complex Bio-Organic Molecules in Aqueous Media. J. Chem. Theory Comput..

[7] Hunt P., Hosseini-Gerami L., Chrien T., Plante J., Ponting D.J., Segall M. (2019). Predicting p*K*_a_ Using a Combination of Semi-Empirical Quantum Mechanics and Radial Basis Function Methods. J. Chem. Inf. Model..

[8] Dutra F.R., de Souza Silva C., Custodio R. (2021). On the Accuracy of the Direct Method to Calculate pKa from Electronic Structure Calculations. J. Phys. Chem. A.

[9] Pereira R.W., Ramabhadran R.O. (2020). pK-Yay: A Black-Box Method Using Density Functional Theory and Implicit Solvation Models to Compute Aqueous pK_a_ Values of Weak and Strong Acids. J. Phys. Chem. A.

[10] Yang Q., Liu Y., Zhang L., Luo S., Cheng J.-P. (2020). Holistic Prediction of the pKa in Diverse Solvents Based on a Machine Learning Approach. Angew. Chem. Int. Ed..

[11] Geremia K.L., Seybold P.G. (2019). Computational Estimation of the Acidities of Purines and Indoles. J. Mol. Model..

[12] Seybold P.G., Shields G.C. (2015). Computational estimation of pK_a_ values. WIREs Comput. Mol. Sci..

[13] Alongi K.S., Shields G.C. (2010). Theoretical Calculations of Acid Dissociation Constants: A Review Article. Ann. Rep. Comput. Chem..

[14] Liptak M.D., Gross K.C., Seybold P.G., Feldgus S., Shields G.C. (2002). Absolute pK_a_ determinations for substituted phenols. J. Am. Chem. Soc..

[15] Seybold P.G. (2012). Quantum Chemical QSPR Estimation of the Acidities and Basicities of Organic Compounds. Adv. Quantum Chem..

[16] Seybold P.G. (2015). Quantum Chemical Estimation of the Acidities of Some Inorganic Oxoacids. Mol. Phys..

[17] Seybold P.G. (2016). Computational Estimation of the Acidities of Some Inorganic Nitrogen Acids. Mol. Phys..

[18] Baldasare C.A., Seybold P.G. (2020). Computational Estimation of the Gas-Phase and Aqueous Acidities of Carbon Acids. J. Phys. Chem. A.

[19] Liao C., Nicklaus M.C. (2009). Comparison of nine programs predicting pKa values of pharmaceutical substances. J. Chem. Inf. Model..

[20] Dearden J.C., Rotureau P., Fayet G. (2013). QSPR prediction of physico-chemical properties for REACH. SAR QSAR Environ. Res..

[21] Gross K.C., Seybold P.G. (2000). Substituent Effects on the Physical Properties and pK_a_ of Aniline. Int. J. Quantum Chem..

[22] Gross K.C., Seybold P.G., Hadad C.M. (2002). Comparison of Different Atomic Charge Schemes for Predicting pKa Variations in Substituted Anilines and Phenols. Int. J. Quantum Chem..

[23] Gross K.C., Hadad C.M., Seybold P.G. (2011). Charge Competition in Halogenated Hydrocarbons. Int. J. Quantum Chem..

[24] Scheiner S., Seybold P.G. (2009). Quantum chemical analysis of the energetics of the anti and gauche conformers of ethanol. Struct. Chem..

[25] Varekova R.S., Geidl S., Ionescu C.-M., Skrehota O., Kudera M., Sehnal D., Bouchal T., Abagyan R., Huber H.J., Koca J. (2011). Predicting pK_a_ Values of Substituted Phenols from Atomic Charges: Comparison of Different Quantum Mechanical Methods and Charge Distribution Schemes. J. Chem. Inf. Model..

[26] Ugur I., Marion A., Parant S., Jensen J.H., Monard G. (2014). Rationalization of the pKa Values of Alcohols and Thiols Using Atomic Charge Descriptors and Its Application to the Prediction of Amino Acid pKa’s. J. Chem. Inf. Model..

[27] Mull H.F., Turney J.M., Douberly G.E., Schaefer H.F. (2021). Kinetic Stability of Pentazole. J. Phys. Chem. A.

[28] Reed A.E., Curtiss L.A., Weinhold F. (1985). Natural population analysis. J. Chem. Phys..

[29] Reed A.E., Curtiss L.A., Weinhold F. (1988). Intermolecular Interactions from a Natural Bond Orbital, Donor-Acceptor Viewpoint. Chem. Rev..

[30] Glendening E.D., Landis C.R., Weinhold F. (2012). Natural Bond Orbital Methods. WIREs Comput. Mol. Sci..

[31] Advanced Chemistry Development, Inc.: Toronto, ON, Canada. https://www.acdlabs.com/.

[32] Katritzky A.R., Ramsden C.A., Joule J.A., Zhdankin V.V. (2010). Handbook of Heterocyclic Chemistry.

[33] Serjeant E.P., Dempsey B. (1979). Ionization Constants of Organic Acids in Aqueous Solution.

[34] (2008). CRC Handbook of Chemistry and Physics.

[35] Dean J.A. (1985). Lange’s Handbook of Chemistry.

[36] Levene P.A., Bass L.W., Simms H.S. (1926). The ionization of pyrimidines in relation to the structure of pyrimidine nucleosides. J. Biol. Chem..

[37] Darnall K.R., Townsend L.B., Robins R.K. (1967). The structure of showdomycin, a novel carbon-linked nucleoside antibiotic related to uridine. Proc. Nat. Acad. Sci. USA.

[38] Privat E.J., Sowers L.C. (1996). A proposed mechanism for the mutagenicity of 5-formyluracil. Mutat. Res..

[39] Handschmacher R.E., Welch A.D. (1956). Microbial studies of 6-azauracil, an antagonist of uracil. Cancer Res..

[40] NIST Chemistry WebBook. http://webbook.nist.gov/chemistry/.

[41] Marenich A.V., Olson R.M., Kelly C.P., Cramer C.J., Truhlar D.G. (2007). Self-consistent reaction field model for aqueous and nonaqueous solutions based on accurate polarized partial charges. J. Chem. Theory Comput..

[42] Cramer C.J., Truhlar D.G. (2008). A Universal Approach to Solvation Modeling. Accts. Chem. Res..

[43] Hunter N.E., Seybold P.G. (2014). Theoretical estimation of the aqueous pK_a_s of thiols. Mol. Phys..

[44] Wessner R.A. (2016). Theoretical Estimation of pK_a_s of Pyrimidines and Related Heterocycles. Master’s Thesis.

[45] Scheiner S., Seybold P.G. (2009).

[46] Gauch H.G. (1993). Prediction, Parsimony and Noise. Am. Sci..

